# A Novel Multi-Modal Flexible Headband System for Sleep Monitoring

**DOI:** 10.3390/bioengineering12101103

**Published:** 2025-10-13

**Authors:** Zaihao Wang, Yuhao Ding, Hongyu Chen, Chen Chen, Wei Chen

**Affiliations:** 1 Center for Intelligent Medical Electronics, School of Information Science and Technology, Fudan University, Shanghai 200433, China; zhwang20@fudan.edu.cn (Z.W.); 22210720117@m.fudan.edu.cn (Y.D.); 2 International Human Phenome Institutes (Shanghai), Shanghai 200433, China; 3 Greater Bay Area Institute of Precision Medicine, Guangzhou 510000, China; chenhongyu@ipm-gba.org.cn; 4 Center for Medical Research and Innovation, Shanghai Pudong Hospital, Shanghai 201203, China; 5 Human Phenome Institute, Fudan University, Shanghai 201203, China; 6 School of Biomedical Engineering, the University of Sydney, Darlington, NSW 2006, Australia

**Keywords:** headband system, multi-modal, sleep monitoring, sleep staging

## Abstract

Sleep monitoring is critical for diagnosing and treating sleep disorders. Although polysomnography (PSG) remains the clinical gold standard, its complexity, discomfort, and lack of portability limit its applicability for long-term and home-based monitoring. To overcome these challenges, this study introduces a novel flexible headband system designed for multi-modal physiological signal acquisition, incorporating dry electrodes, a six-axis inertial measurement unit (IMU), and a temperature sensor. The device supports eight EEG channels and enables wireless data transmission via Bluetooth, ensuring user convenience and reliable long-term monitoring in home environments. To rigorously evaluate the system’s performance, we conducted comprehensive assessments involving 13 subjects over two consecutive nights, comparing its outputs with conventional PSG. Experimental results demonstrate the system’s low power consumption, ultra-low input noise, and robust signal fidelity, confirming its viability for overnight sleep tracking. Further validation was performed using the self-collected HBSleep dataset (over 184 h recordings of the 13 subjects), where state-of-the-art sleep staging models (DeepSleepNet, TinySleepNet, and AttnSleepNet) were applied. The system achieved an overall accuracy exceeding 75%, with AttnSleepNet emerging as the top-performing model, highlighting its compatibility with advanced machine learning frameworks. These results underscore the system’s potential as a reliable, comfortable, and practical solution for accurate sleep monitoring in non-clinical settings.

## 1. Introduction

Sleep is a vital physiological process that occupies nearly one third of the human lifespan and is fundamental to health and cognition [1]. Yet in modern society, chronic sleep problems affect almost one third of adults worldwide, with insomnia and obstructive sleep apnea among the most prevalent [2,3]. These disorders increase the risk of cardiovascular and metabolic disease, impair cognitive performance, and contribute to psychiatric comorbidities, placing a heavy burden on healthcare systems. Beyond individual health, insufficient and poor-quality sleep also generates substantial societal costs through lost productivity, traffic accidents, and occupational errors [4]. Together, these facts underscore the urgent need for accessible and reliable sleep assessment.

Polysomnography (PSG) remains the gold standard for diagnosing sleep disorders, but its practical constraints limit scalability. PSG requires specialized facilities, complex setup with dozens of electrodes and sensors, and overnight supervision. This leads to high cost, discomfort, and disruption of natural sleep, and it hampers longitudinal or home-based monitoring [5,6,7,8,9]. As a result, PSG cannot feasibly support large-scale or long-term surveillance of sleep health [10].

In response, a growing body of research has explored wearable and wireless sleep monitoring systems. However, despite technical progress, current devices share recurring shortcomings [11,12]. Current sleep monitoring technologies can be broadly categorized into systems that exclude or incorporate electroencephalography (EEG). Devices relying solely on auxiliary signals such as blood oxygen saturation, motion, or acoustics (e.g., Smartwatches [13], EarSleep [14]) are convenient and low-cost, but their accuracy is fundamentally constrained by the absence of EEG, the gold standard for sleep staging. In contrast, EEG-based systems (e.g., Cognionics headset [15], BioWolf [16], headband-type devices [17,18]) offer greater reliability, yet their rigid electrodes and hard enclosures markedly compromise comfort, limiting their applicability for long-term sleep monitoring and confining their use primarily to brain–computer interface or motor imagery research [15,16]. More recently, forehead- or patch-based EEG solutions [19,20] have been proposed to improve wearability; however, these devices capture only frontal activity while neglecting critical signals from hair-covered regions such as the occipital and temporal lobes. These limitations underscore the need for flexible EEG technologies that reconcile comfort with comprehensive signal acquisition for practical sleep monitoring applications.

This work proposes a flexible headband designed to overcome these limitations. The system integrates eight dry EEG electrode channels distributed across occipital, temporal, and frontal sites, together with inertial and temperature sensing, to capture richer sleep-related signals. The lightweight, fabric-based construction avoids rigid elements at the back and sides of the head, supporting comfort across sleeping postures. Wireless transmission and low power consumption enable overnight home use. By addressing capability and usability in a single platform, this headband aims to provide a feasible step toward scalable, minimally disturbing sleep monitoring.

## 2. Design of the Novel Flexible Headband and Experimental Setup

### 2.1. Headband Design

**Figure 1:**
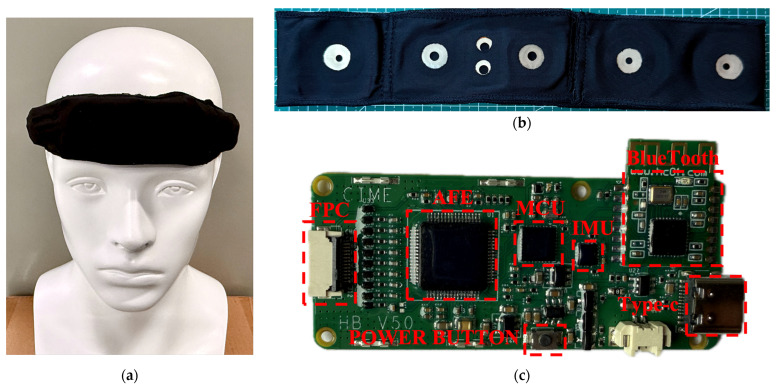
The wearing effect of the novel flexible headband system on a mannequin, along with the key upgrades and modifications: (**a**) the headband being worn by a head model; (**b**) the Lycra cotton band; (**c**) the redesigned acquisition circuit board.

The headband system is an upgraded version of a previously developed basic headband prototype [21]. It evolves from a simple strap-based design to an integrated headband, offering significantly improved convenience in wearing. In addition, the electrode interface and certain functions of the acquisition board have been enhanced. Compared with the earlier functional prototype, the new headband system shows substantial improvements in both appearance and wearing comfort, making it more user-friendly. Since the signal acquisition performance of the previously developed headband prototype had already been validated against the gold-standard polysomnography (PSG) system, verification tests of the headband were omitted in this study [21]. Figure 1 illustrates the wearing effect of the novel flexible headband system on a mannequin, along with the key upgrades and modifications.

The headband strap plays a critical role in ensuring both the comfort and stability of the system. The material must be not only elastic and skin-friendly but also capable of securing the electrode connectors. As shown in Figure 1b, Lycra cotton was ultimately selected. This fabric offers good elasticity and is commonly used in infant clothing due to its skin-friendly properties [22,23,24]. An integrated cutting design was adopted to maintain the structural integrity of the headband. The plastic casing was removed, and multiple fabric layers were sewn at the forehead area to form pouches that directly house the circuit board and lithium battery, further enhancing the headband’s flexibility and wearing comfort.

Figure 1c shows the redesigned acquisition circuit board, where the electrode interface uses an FPC (Flexible Printed Circuit) connector, ensuring reliable connectivity while allowing easy disassembly. Since the headband is made from one-piece Lycra cotton and all electrode connectors and wires are embedded inside, the electrodes and the circuit board can be separated, allowing the band itself to be independently washed. Additionally, because toggle switches were inconvenient to use when placed between the rear straps, a button-type power switch was introduced: pressing for 3 s powers on the system, and pressing for 5 s powers it off.

### 2.2. Power Consumption Test

For wearable systems, power consumption is a critical performance metric that directly affects feasibility and user experience. Therefore, the power consumption of the wearable dry-electrode EEG acquisition system was tested. The test equipment used was the Keithley 2612B dual-channel system source meter from the United States. The output voltage was set to the lithium battery’s rated voltage of 3.7 V, with a current sampling interval of 25 ms.

To optimize system power consumption, two different data transmission strategies were designed in addition to selecting low-power chips: single-packet transmission and multi-packet transmission. In single-packet transmission, data is packaged and sent to the host computer via Bluetooth immediately after each sampling. In multi-packet transmission, multiple samples (50 samples) are packaged together before being sent via Bluetooth. Consequently, power consumption tests were conducted separately for the two transmission strategies.

### 2.3. Input Noise Test

Input noise is a critical parameter for bioelectrical acquisition systems, especially for capturing weak EEG signals. Therefore, input noise testing was conducted on the system. First, all input electrodes were short-circuited, and normal data acquisition was performed. The collected data was received and saved by the host computer. Ideally, because of the short-circuited inputs, the recorded data should be zero; however, due to noise interference, the actual acquired signals are non-zero, representing the system’s input noise. Since the main frequency range of EEG signals is 0.3–35 Hz, the collected data was uniformly processed with filtering: a 0.3 Hz high-pass filter followed by a 35 Hz low-pass filter.

### 2.4. Overnight Sleep Monitoring Experiment

The novel flexible headband system is designed for overnight sleep monitoring. To validate its practical performance in this context, an overnight sleep monitoring experiment was conducted involving healthy participants. The study was approved by the Fudan University Ethics Committee (Approval No.: FE231711) following a biomedical research ethical review process. A total of 13 subjects participated in the formal experiment, including 8 males and 5 females, aged between 22 and 33 years. Each subject completed two nights of sleep experiments.

**Figure 2:**
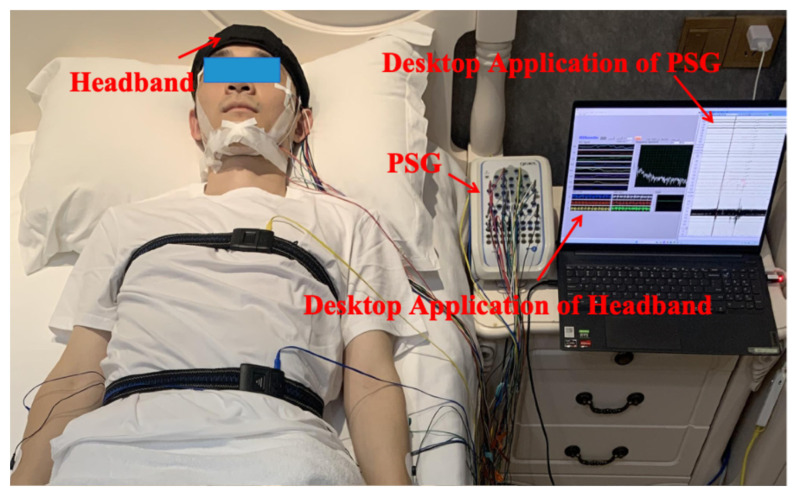
Photograph of the sleep monitoring experimental setup.

The experiments were conducted in a small testing room. In addition to wearing the flexible headband system, participants also wore a PSG device following AASM standards to serve as the gold standard for sleep labeling. Figure 2 shows a photograph of a subject during the sleep experiment data collection.

### 2.5. User Survey on Convenience and Comfort

During the entire sleep experiment, in addition to recording the participants’ basic information, the experiment duration and the subjects’ comfort level with the headband were also documented. The experiment duration mainly compared the time required to put on and take off the headband versus the PSG equipment. Headband comfort was assessed using a Visual Analog Scale (VAS), a commonly used tool to evaluate the intensity of subjective sensations such as pain, fatigue, or emotions [25,26].

The VAS involves participants marking their perceived intensity on a line segment, typically ranging from “no discomfort/no pain” to “worst discomfort/unbearable.” The length of the scale is usually set according to the assessment needs, commonly 10 cm. This scale is simple, easy to understand, and allows quantification of subjective feelings by measuring the distance from the starting point to the mark [27].

**Figure 3:**
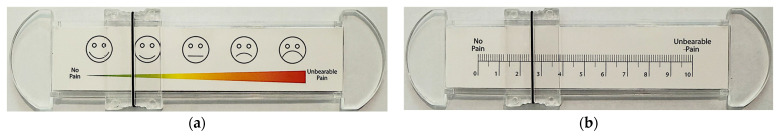
The VAS Rating Scale: (**a**) the emoticon side of the VAS Rating Scale; (**b**) the 10 cm millimeter scale side of the VAS Rating Scale.

Figure 3 shows the VAS rating scale used in this experiment: one side displays emoticons (Figure 3a), while the other side has a 10 cm millimeter scale (Figure 3b). During the test, the experimenter faces the emoticon side towards the participant and asks the relevant questions. The participant then moves a cursor along the scale to indicate their feeling. The experimenter records the cursor’s position by reading the corresponding millimeter value on the reverse side. The comfort scale was divided as follows: Very comfortable (0–20 mm), Comfortable (21–40 mm), Uncomfortable (41–60 mm), Annoying (61–80 mm), Unbearable (81–100 mm).

### 2.6. Reliability Test of the Comfort Scale

The reliability of the comfort rating scale was assessed to ensure its consistency and stability under varying conditions. It is essential in scientific research, psychology, education, medicine, engineering, and data analysis to ensure the reliability of experimental data and measurement instruments. There are typically two main approaches to reliability testing: external consistency and internal consistency. A common method for external consistency is test–retest reliability, where the same participant completes the scale twice under similar conditions, and the results are compared to determine consistency. For internal consistency, the most widely used method is the Cronbach’s alpha coefficient [28]. Cronbach’s alpha (α) ranges from 0 to 1 and is interpreted as follows: α ≥ 0.9 indicates excellent internal consistency; 0.8 ≤ α < 0.9 indicates good internal consistency; 0.7 ≤ α < 0.8 indicates acceptable; 0.6 ≤ α < 0.7 indicates questionable; 0.5 ≤ α < 0.6 indicates poor; α < 0.5 indicates unacceptable. Equation (1) shows the formula for calculating Cronbach’s alpha.

(1) $$\alpha =\frac{K}{K-1}(1-\frac{\sum_{i=1}^{K}\sigma_{i}^{2}}{\sigma_{T}^{2}})$$

In this study, K represents the number of items in the questionnaire; $\sigma_{i}^{2}$ is the variance of the i-th item; and $\sigma_{T}^{2}$ is the variance of the total score across all items. Due to the influence and adaptive changes between the two experimental sessions, external consistency testing (e.g., test–retest reliability) was deemed unsuitable for evaluating the reliability of the comfort scale in this experiment. Therefore, Cronbach’s alpha coefficient was selected to assess internal consistency.

### 2.7. Statistical Analyses

Statistical analyses were conducted to evaluate the potential effects of night and gender on participants’ subjective comfort scores, with the aim of exploring whether comfort varied systematically across repeated nights or between male and female participants. A paired-sample t-test was used to compare scores between the two nights (within-subject factor: Night), while an independent-samples t-test assessed gender-related differences based on each participant’s mean score across nights (between-subject factor: Gender). The significance level was set at 0.05. Normality was checked with Shapiro–Wilk tests and homogeneity of variance with Levene’s test. When assumptions were violated, Wilcoxon signed-rank or Mann–Whitney U tests were additionally performed. Effect sizes (Cohen’s dz/gz or d/g) were reported. These tests provided an initial indication of whether night-to-night variation or gender differences might influence comfort, thereby offering preliminary insights to guide more comprehensive assessments in future studies.

### 2.8. Validation Using Existing Sleep Staging Models

Current sleep monitoring research primarily focuses on sleep stage classification. Therefore, after collecting sleep data using the headband system, we employed several existing sleep staging models to train and predict sleep stages, thereby further validating the system’s effectiveness in sleep monitoring applications. EEG signals serve as the core basis for sleep stage classification. Accordingly, we selected three widely adopted single-channel EEG models for verification: DeepSleepNet [29], TinySleepNet [30], and AttnSleepNet [31]. EEG signals from each of the eight channels were input into these models, and performance was evaluated using metrics such as accuracy, F1 score, and Cohen’s Kappa coefficient.

Model training was conducted using PyTorch 1.4.0 on Python 3.6 with an NVIDIA GeForce RTX 1080Ti GPU. The batch size was set to 128, learning rate to 1 × 10^−4^, maximum epochs to 100, with a weight decay of 1 × 10^−4^ in the Adam optimizer, L2 regularization, and a dropout probability of 0.5. Detailed hyperparameters are shown in Table 1.

To maintain data integrity, the stored dataset comprised raw, unprocessed signals. Prior to input into the models, the data underwent preprocessing including filtering, downsampling, and segmentation. As sleep-related physiological signals are mainly distributed between 0.3 Hz and 35 Hz and are susceptible to power line interference (50 Hz), a sixth-order 49–51 Hz Butterworth notch filter was first applied to remove such noise. Then, a 35 Hz low-pass and a 0.3 Hz high-pass Butterworth filter were applied to eliminate high-frequency interference and low-frequency drift, respectively. To minimize data volume and computational load while retaining essential signal characteristics, the sampling rate was reduced from 250 Hz to 100 Hz. This not only eased computational load but also better matched the input format of deep learning models. For compatibility with AASM sleep staging standards, continuous signals were segmented into fixed 30 s epochs. Each epoch was assigned a sleep stage label, ensuring one-to-one correspondence between signal and label.

## 3. Results and Discussion

### 3.1. System Power Consumption

**Figure 4:**
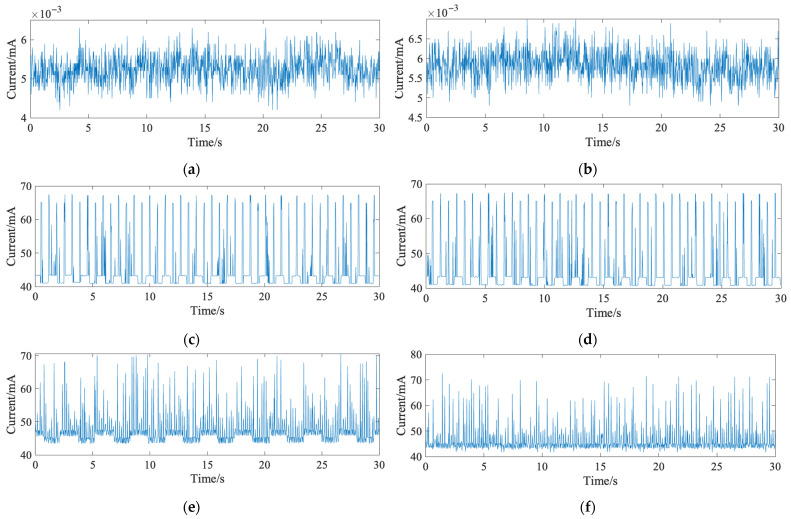
The current waveforms of the system under different transmission strategies: (**a**) standby current waveform in single-packet transmission mode; (**b**) standby current waveform in multi-packet transmission mode; (**c**) operating current waveform in single-packet transmission mode; (**d**) operating current waveform in multi-packet transmission mode; (**e**) data transmission current waveform without signal detection in single-packet mode; (**f**) data transmission current waveform without signal detection in multi-packet mode.

Figure 4 shows the current waveforms of the system under different transmission strategies. Specifically, Figure 4a,b illustrate the standby current for single-packet and multi-packet transmission, respectively. Figure 4c,d show the current under Bluetooth disconnected status for single-packet and multi-packet transmission, respectively. Figure 4e,f present the current waveforms during Bluetooth connection and data transmission for single-packet and multi-packet modes, respectively. The current waveforms under standby and disconnected states were nearly identical for both strategies since data transmission was not involved. During transmission, multi-packet mode exhibited larger waveform amplitude but less frequent fluctuations, while single-packet mode was more stable but slightly higher on average.

For further analysis, feature parameters were extracted from the waveforms, with the results shown in Table 2. The multi-packet mode had a higher peak-to-peak fluctuation (31.0 mA) compared to the single-packet mode (27.3 mA), but its average current consumption was lower (45.9 mA vs. 47.3 mA). Therefore, multi-packet mode was selected for the final system. Based on an 8 h sleep period, only a 370 mAh battery is required to support full-night operation.

### 3.2. Input Noise

**Figure 5:**
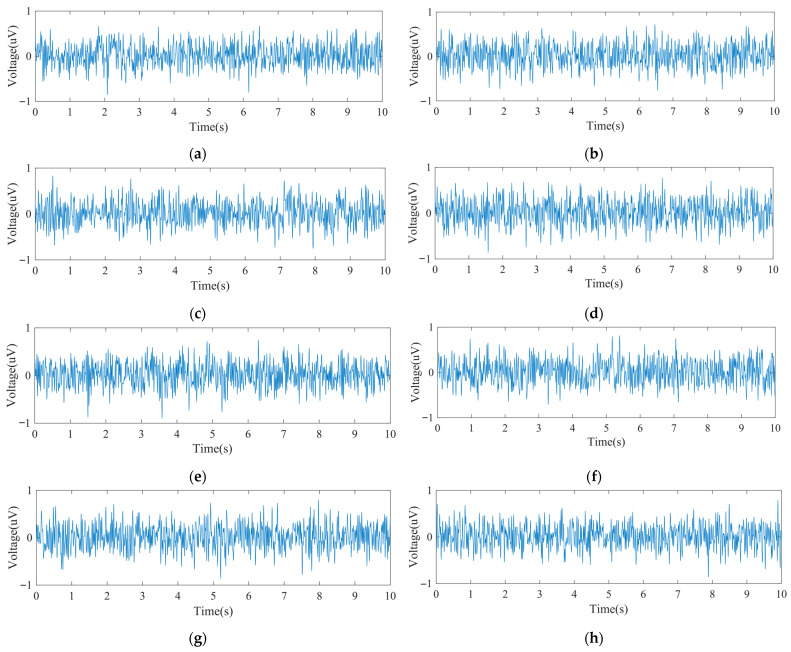
The input noise: (**a**) Channel 1 input noise waveform; (**b**) Channel 2 input noise waveform; (**c**) Channel 3 input noise waveform; (**d**) Channel 4 input noise waveform; (**e**) Channel 5 input noise waveform; (**f**) Channel 6 input noise waveform; (**g**) Channel 7 input noise waveform; (**h**) Channel 8 input noise waveform.

Figure 5 shows the input noise waveforms for the system’s eight EEG channels, all fluctuating within ±0.8 µV. Table 3 summarizes the noise characteristics, including peak values, mean, and RMS. The average input noise ranged between 0.015 µV and 0.022 µV, with the largest peak-to-peak value being 1.66 µV. Since sleep-related EEG amplitudes generally exceed 10 µV, the system’s input noise performance is adequate for EEG acquisition.

### 3.3. Evaluation of Headband Convenience and Comfort

#### 3.3.1. Donning and Doffing Efficiency

In this experiment, the PSG system required assistance from one or two experimenters for both donning and doffing procedures. In contrast, the headband was designed for independent use, allowing subjects to wear and remove it autonomously as needed. The donning and doffing durations for both the PSG and the headband were recorded in minutes, rounded up to the nearest whole minute.

**Figure 6:**
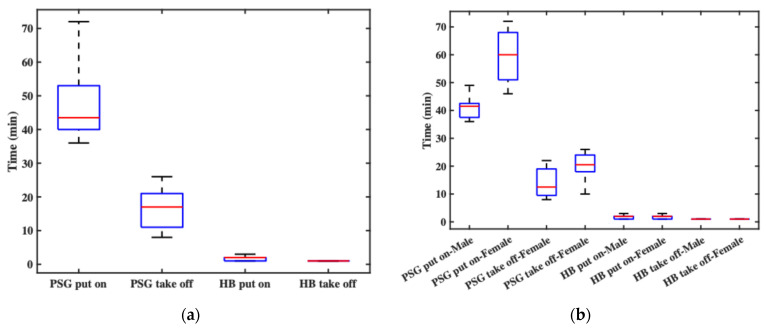
The recorded durations: (**a**) the overall statistics of donning and doffing times; (**b**) the donning and doffing times by gender.

Figure 6 presents box plots of the recorded durations. Figure 6a shows overall statistics: the PSG donning time ranged from 36 to 72 min (a twofold difference), influenced by the experimenters’ experience and the participants’ characteristics (e.g., hair length) and cooperation. The average PSG donning time was 48 min. In contrast, the headband’s donning time ranged from 1 to 3 min, with an average of 1.8 min, mainly due to brief system debugging. This clearly demonstrates the headband’s superior donning efficiency. For doffing, the PSG required 8 to 26 min, averaging 16 min, largely affected by the participant’s condition. The headband, however, required less than one minute to remove. Figure 6b breaks down the results by gender. For male participants, PSG donning time ranged from 36 to 49 min, with an average of 40.9 min. For female participants, the time ranged from 46 to 72 min, averaging 59.4 min. In terms of PSG doffing, male subjects ranged from 8 to 22 min (average 13.9 min), while female subjects ranged from 10 to 26 min (average 19.5 min). The time required to don the headband was comparable between genders, ranging from 1 to 3 min, with mean durations of 1.9 min for males and 1.8 min for females. Doffing times for the headband were consistently under 1 min across all participants. These results indicate that both donning and doffing of the PSG system took significantly longer for female subjects, primarily due to longer hair. In contrast, the headband system showed no significant gender-based differences, indirectly confirming that hair length had minimal impact on its usability.

#### 3.3.2. Comfort Assessment

**Figure 7:**
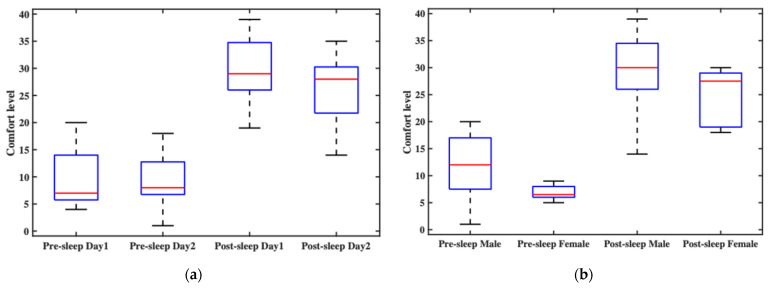

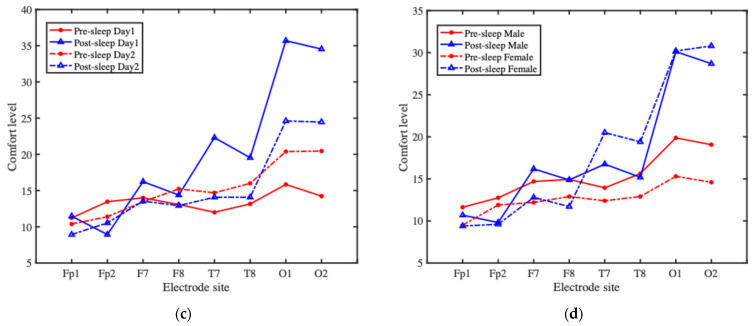
The overall wearing comfort of the headband system and comfort ratings at each electrode site: (**a**) the participants’ overall comfort ratings before and after sleep across two consecutive nights; (**b**) the comfort ratings between male and female participants; (**c**) the average comfort ratings at each electrode site over time; (**d**) the average comfort ratings at each electrode site, grouped by gender.

In this experiment, comfort level was statistically analyzed from two perspectives: time and gender. Figure 7a shows participants’ overall comfort ratings before and after sleep across two consecutive nights. The pre-sleep comfort levels generally fell within the “relatively comfortable” range, though slight variations were observed between the two nights, primarily due to the initial experience on the first night. Post-sleep comfort was slightly lower than pre-sleep but still remained within the “comfortable” range. Notably, post-sleep comfort on the second night was better than on the first, indicating that participants needed some time to adapt to wearing the headband throughout the night. Figure 7b compares comfort ratings between male and female participants. Regardless of whether the ratings were collected before or after sleep, female participants consistently reported higher comfort levels than male participants. This may be attributed to the fact that most female subjects had longer hair, which could have helped cushion the contact points and improve overall wearing comfort.

To further analyze the factors affecting comfort, in addition to assessing overall headband comfort, we also conducted a detailed evaluation of the comfort level at each electrode site. Figure 7c presents the comfort ratings at each electrode site over time. Pre-sleep electrode comfort on the second night was generally rated lower than on the first night, whereas post-sleep comfort was rated higher on the second night compared to the first. Overall, post-sleep comfort tended to be lower than pre-sleep comfort, indicating a decline in comfort during overnight wear. This post-sleep discomfort likely influenced participants’ perception and rating of pre-sleep comfort on the following day, leading to lower reported comfort scores. Figure 7d shows comfort ratings at each electrode site, grouped by gender. For both male and female participants, the most significant variation in comfort occurred at the O1 and O2 electrode sites, while the comfort ratings at other electrode sites showed relatively minor changes. Male participants reported slightly lower pre-sleep comfort at the electrode sites compared to females; however, post-sleep comfort ratings between the two groups were similar, though with noticeable fluctuations.

The results suggest that wearing the headband overnight involves an adaptation process. After the first night, participants generally reported improved comfort on the second night. In summary, all electrode sites fell within the “relatively comfortable” range before sleep. After sleep, most electrodes remained in that range, with the exception of O1 and O2, which were rated as “comfortable.” The two electrodes are placed in the occipital region, which is frequently subjected to pressure during supine sleep, potentially contributing to the marked decrease in comfort reported at these sites.

#### 3.3.3. Reliability Test of the Comfort Scale

In the comfort rating scale, the overall comfort levels before and after sleep were treated as two independent items. In addition, the comfort levels of each electrode site before and after sleep were treated as 16 independent items, resulting in a total of 18 items. The responses from the two experimental nights were treated as two separate datasets for analysis. The calculated Cronbach’s alpha coefficient for the comfort scale was 0.9345, indicating excellent internal consistency and demonstrating that the scale provides reliable and consistent results.

#### 3.3.4. Statistical Analyses

Table 4 shows a comparison of comfort ratings between the first and second nights across overall headband and electrode sites. Between the first and second nights, overall comfort improved significantly (t(12) = 2.49, *p* = 0.028; W = 14.5, *p* = 0.033, dz = 0.69, gz = 0.65). At the electrode level, significant increases were observed at T7 (t(12) = 2.59, *p* = 0.024; W = 0, *p* = 0.003, dz = 0.72), O1 (t(12) = 3.23, *p* = 0.007; W = 4, *p* = 0.002, dz = 0.90), and O2 (t(12) = 4.41, *p* < 0.001; W = 0, *p* < 0.001, dz = 1.22), with effect sizes ranging from medium-to-very large, while no significant changes were found at frontal electrodes (Fp1, Fp2, F7, F8) or T8 (all *p* > 0.05); although Shapiro–Wilk tests indicated deviations from normality for some sites (e.g., Fp1, F8, T7, *p* < 0.01), the robust improvements at T7, O1, and O2 were consistent across both parametric and nonparametric analyses.

Table 5 is the comparison between male and female participants indicated no significant differences in comfort rating. For overall headband comfort, the independent-samples t-test showed no significant difference (t(24) = 1.69, *p* = 0.105), consistent with the Mann–Whitney U test (U = 111.5, *p* = 0.101). Although not statistically significant, the effect size was moderate (d = 0.63, g = 0.61), suggesting a potential trend. At the electrode level, no significant gender differences were found across Fp1, Fp2, F7, F8, T7, T8, O1, and O2 (t-tests: *p* = 0.327–0.989; Mann–Whitney U: *p* > 0.05). Effect sizes were small or negligible (|d| < 0.41). Shapiro–Wilk tests indicated deviations from normality at some sites (e.g., Fp1, F8), but Levene’s test results were non-significant (*p* > 0.05), indicating homogeneity of variance.

### 3.4. Algorithm Evaluation Using the Custom Dataset

#### 3.4.1. Construction of the HBSleep Dataset

A proprietary sleep dataset named HBSleep was constructed based on data collected from the headband system and sleep stage annotations derived from PSG. A total of 13 healthy participants were included in the study, consisting of 8 males and 5 females, with ages ranging from 22 to 33 years. Each participant underwent two nights of sleep monitoring. However, due to specific issues encountered during some sessions, such as significant body movement at night causing PSG signal degradation or electrode detachment, certain nights’ data could not be labeled by clinicians and were excluded from the final dataset.

As a result, 24 nights of valid sleep data were collected, with a total duration of approximately 184.3 h. The dataset consists of raw data files and corresponding annotation files, both stored in .txt format. Raw data files are named using the participant ID (e.g., P01.txt), and annotation files are named with the participant ID followed by _annotations (e.g., P01_annotations.txt). Each raw data file contains: 8 channels of EEG signals, 3-axis accelerometer data, 3-axis gyroscope data and ambient temperature readings.

The annotation files contain sleep stage labels aligned with the corresponding raw data. These labels were manually scored by certified sleep technicians using PSG data, following the AASM standard, and then converted to align with the headband data timeline. This dataset provides a rich resource for sleep analysis research, wearable algorithm development, and model validation.

#### 3.4.2. Input Data Description

After preprocessing, the proprietary dataset HBSleep consists of a total of 22,120 epochs. The distribution across sleep stages is as follows: Wake stage has 4353 epochs (19.68%); N1 stage has 1456 epochs (6.58%); N2 stage has 9855 epochs (44.55%); N3 stage has 3116 epochs (14.09%); REM stage has 3340 epochs (15.10%). Table 6 presents the detailed statistics for each sleep stage. Because the baseline models accept only single-channel input, each EEG channel was saved separately in .npz format for training and testing.

#### 3.4.3. Baseline Algorithm Evaluation and Analysis

The quality of the HBSleep dataset was evaluated by applying baseline classification models to perform sleep stage classification. Performance metrics such as accuracy, precision, recall, F1-score, and Cohen’s kappa coefficient were calculated to assess the dataset’s usability and to validate the feasibility of using the headband system for sleep monitoring. Table 7 presents the average results and standard deviations obtained from the EEG data of all 8 channels across three baseline models. These metrics reflect the models’ classification performance and the consistency of signal quality across channels. The evaluation demonstrates that the data collected by the headband system supports reliable sleep staging, confirming its potential for use in non-clinical, at-home sleep monitoring applications.

Among the three models, AttnSleepNet achieved the best overall performance, with the highest average metrics across channels: accuracy (75.81%), precision (64.58%), recall (64.73%), F1-score (62.70%), and Cohen’s kappa (65.52%). These results indicate that AttnSleepNet offers superior classification capability and robustness when applied to the proprietary HBSleep dataset. TinySleepNet ranked second, slightly outperforming DeepSleepNet across all key metrics, including average accuracy, precision, recall, F1-score, and kappa. This suggests that lightweight models like TinySleepNet may have advantages in scenarios involving relatively small-scale datasets. Furthermore, the average kappa scores across channels for all three models fall within the range of “substantial agreement” (0.61–0.80) according to the Landis & Koch scale, demonstrating that the label quality of the HBSleep dataset is sufficient to support reliable algorithm training and evaluation.

**Figure 8:**
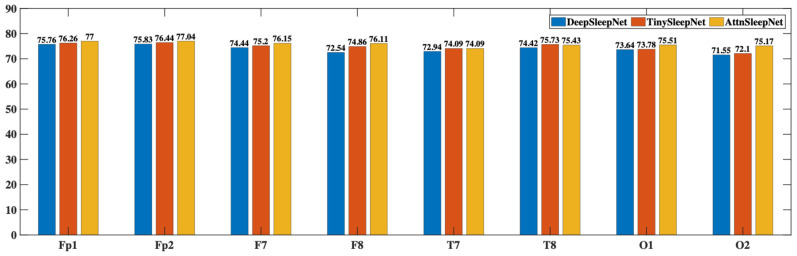
Accuracy of EEG Data from Each Channel Across Different Models.

Figure 8 shows the accuracy performance of each channel’s data across different models. It is clear that AttnSleepNet achieves the best accuracy on every channel, with the highest single-channel accuracy reaching 77.04% at the Fp2 channel. Following that is TinySleepNet, with its highest accuracy also at the Fp2 channel at 76.44%. DeepSleepNet shows relatively lower accuracy, with its highest at Fp2 channel of 75.83%. These results suggest that prefrontal EEG channels (Fp1 and Fp2) provide the most reliable signals, likely due to fewer motion artifacts during sleep. In contrast, occipital channels (e.g., O1 and O2) showed slightly lower accuracy, possibly due to pillow contact or head movements during sleep, which interfere with signal quality.

**Figure 9:**
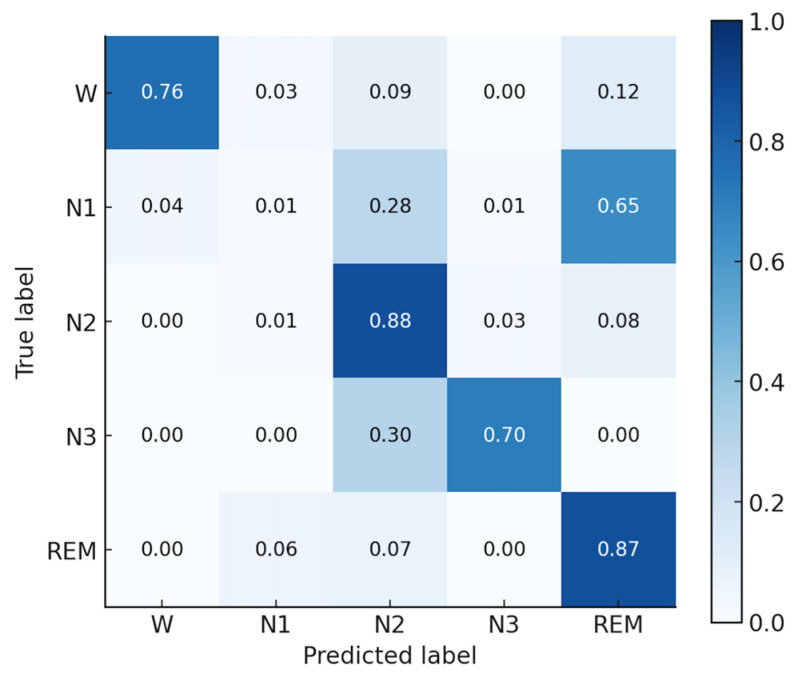
Normalized confusion matrix of the AttnSleepNet model using the Fp2 channel.

Since the Fp2 channel achieved the highest classification accuracy (77.04%) with the AttnSleepNet model, this result was selected for visualization of the confusion matrix. Figure 9 shows the normalized confusion matrix of the AttnSleepNet model using the Fp2 channel. The model achieves the best performance in N2 (88%) and REM (87%) stages, highlighting its strong discriminative ability in recognizing major sleep phases. The W stage attains a reasonable accuracy of 76%, though some samples are misclassified as REM (12%) or N2 (9%). For the N3 stage, the accuracy reaches 70%, with around 30% of the samples confused with N2, indicating difficulties in distinguishing deep sleep from light-to-intermediate sleep. In contrast, the N1 stage shows the weakest performance, with only 1% correctly identified, while most samples are misclassified as N2 (28%) and REM (65%). Overall, the AttnSleepNet model on the Fp2 channel demonstrates strong recognition ability for major sleep stages, but struggles considerably in identifying the N1 stage.

## 4. Discussion

The statistical results demonstrated that comfort perception was not static but dynamically changed with repeated use. Second-night comfort was significantly higher than first-night comfort, suggesting a clear adaptation effect to the wearable headband. This finding has practical implications: with continued use, participants became more accustomed to the device, resulting in reduced discomfort and potentially higher compliance in long-term sleep monitoring. The most pronounced improvements were observed in the occipital region (O1 and O2), with large to very large effect sizes. This suggests that posterior electrode sites benefited most from habituation, possibly due to adjustments in sleep posture, reduced sensitivity to pressure, or better electrode-skin adaptation over time. The significant improvement at T7 further indicates that lateral regions may also undergo adaptation. By contrast, frontal electrodes (Fp1, Fp2, F7, F8) did not show significant changes across nights. This may be attributed to the absence of additional mechanical pressure on the frontal region during normal sleep, which contributes to higher comfort and more stable outcomes. Regarding gender differences, no significant effects were observed for overall or site-specific comfort ratings. Despite a moderate effect size trend in overall headband comfort, statistical tests did not confirm gender differences. This consistency across electrode sites indicates that the current design provides balanced adaptability across male and female users, reducing potential gender-related biases in comfort. Nevertheless, the modest sample sizes may limit statistical power. Moreover, comfort evaluation was subjective and might have been influenced by individual factors such as hairstyle, scalp sensitivity, and personal tolerance to pressure. Future research should expand to larger and more diverse populations and incorporate objective measures of electrode-skin interface.

Baseline model testing on the self-collected HBSleep dataset demonstrated the feasibility of automatic staging with dry electrodes. The attention-based model (AttnSleepNet) achieved the highest mean accuracy (about 75.8%) and kappa (0.6552) with single-channel input, while the best-performing channel (Fp2) reached 77.04% accuracy, confirming that dry-electrode signals are sufficient for deep learning-based sleep staging, and that attention mechanisms are particularly well-suited for this data. The recognition performance of the N1 stage is relatively poor, with a recall of only 1%. This can be attributed to two factors: (i) the limited number of N1 samples, which prevents the model from sufficiently learning its distinctive features; and (ii) the N1 stage has a short duration and shares highly similar signal characteristics with N2 and REM, making it prone to misclassification. As shown in the confusion matrix in Figure 9, approximately 28% of N1 samples are misclassified as N2 and about 65% are misclassified as REM, which is consistent with the above explanation. Future algorithm development will also prioritize the design of dedicated sleep staging models for this system. In particular, incorporating attention mechanisms may allow the model to dynamically focus on informative EEG segments and multimodal features, thereby improving classification accuracy and robustness in real-world settings.

Performance was slightly lower than that reported in public PSG datasets, which use gel or cup electrodes in controlled clinical environments. In contrast, our recordings were acquired at home with dry electrodes, introducing motion and contact noise. This trade-off is offset by significant improvements in comfort and wearability, which are critical for large-scale or longitudinal studies. Channel-wise differences were observed: prefrontal electrodes (Fp1 and Fp2) outperformed occipital electrodes (O1 and O2), likely due to pillow contact and pressure artifacts, indicating that electrode placement and pressure distribution are important factors for future designs.

The comparison summarized in Table 8 highlights several important distinctions across systems. In terms of channel count, research and commercial headsets such as Cognionics headset provide higher-density EEG, whereas most wearable prototypes for sleep monitoring employ fewer channels (1–6), typically positioned outside hair-covered regions. Input noise is generally not specified in some systems, while commercial devices report values below 1 μV RMS, and the proposed system achieves 0.24 μV RMS, indicating superior signal fidelity. Electrode type also varies considerably: wet electrodes (BioWolf) allow stable recordings but reduce wearability, while dry electrodes (Lin 2017 [20], Wang 2024 [17], Cognionics) and patch-based designs (Kwon 2023 [19]) improve user comfort and support long-term monitoring. Regarding multimodal capability, only a subset of systems integrates additional signals (e.g., EOG, EMG), which correlates with their ability to perform sleep staging and apnea detection. Flexibility further differentiates systems, as patch-based and headband devices generally incorporate flexible substrates, unlike rigid headsets. Finally, plastic housings are common in commercial devices but absent in soft patch-based systems, which contributes to higher comfort during sleep. The proposed work demonstrates that combining multimodal integration with flexible dry electrodes can bridge this gap, suggesting a promising pathway toward user-friendly and minimally disturbing sleep monitoring solutions.

However, several limitations must be acknowledged: the small and homogeneous sample, the absence of concurrent PSG validation, the lack of long-term adherence data, and the preliminary nature of the comfort assessment, which was limited to short-term ratings without follow-up. In addition, the statistical validation of staging performance remains underdeveloped, as more rigorous analyses (e.g., mixed-effects models, confidence intervals, and bias assessments) are needed to substantiate the findings.

## 5. Conclusions

This study presents the design, implementation, and preliminary validation of a novel multi-modal flexible headband system for sleep monitoring. Relative to conventional PSG systems, the headband is expected to provide improvements in comfort, ease of use, and applicability to home-based and long-term monitoring. It supports multi-modal signal acquisition, including EEG, motion, and temperature, while maintaining low power consumption and low noise, which are important for overnight use.

User experiments with a limited cohort of 13 healthy subjects suggest that the headband can be worn and removed quickly and comfortably, with minimal impact from gender or hair type. A custom-labeled sleep dataset (HBSleep) was constructed, and three state-of-the-art sleep staging models were evaluated on it. While the classification results remain modest, they provide an initial indication that the signals obtained are usable for exploratory automatic staging.

In summary, this multi-modal flexible headband system demonstrates feasibility as a wearable sleep monitoring tool and provides a foundation for further studies. Future studies will need to include larger and more diverse populations, concurrent PSG comparisons, extended monitoring periods, comprehensive comfort assessments, and a more rigorous statistical framework to establish the generalizability and clinical utility of the system, as well as incorporate detailed analyses based on the characteristics of the proposed system to design tailored sleep staging algorithms that improve classification accuracy.

## Figures and Tables

**Table 1:** Hyperparameter settings.

Hyperparameter	Value
Batch size	128
Learning rate	10^−4^
Weight decay	10^−4^
Dropout	0.5
Epochs	100

**Table 2:** Current (mA) characteristics under different conditions.

Mode	State	Max	Min	Vp-*p*	Mean
Multi	Standby	7 uA	4.8 uA	2.2 uA	5.8 uA
	Disconnect	67.5	40.6	26.9	45.1
	Transmission	72.6	41.6	31.0	45.9
Single	Standby	6.3 uA	4.2 uA	2.1 uA	5.2 uA
	Disconnect	67.6	40.8	26.8	45.1
	Transmission	70.6	43.3	27.3	47.3

**Table 3:** Feature parameters (uV) of input noise waveforms for the system’s 8 channels.

Channel	Max	Min	Vp-*p*	Mean	RMS
CH1	0.67	−0.85	1.52	0.019	0.23
CH2	0.72	−0.77	1.49	0.022	0.24
CH3	0.83	−0.75	1.58	0.016	0.24
CH4	0.77	−0.86	1.63	0.022	0.25
CH5	0.74	−0.90	1.64	0.022	0.23
CH6	0.81	−0.71	1.52	0.015	0.24
CH7	0.8	−0.86	1.66	0.018	0.24
CH8	0.79	−0.86	1.65	0.017	0.23

**Table 4:** Comparison of comfort ratings between the first and second nights across overall headband and electrode sites.

Comparison	t (df)	*t*-Test *p*	95% CI	Effect	Shapiro *p*	Wilcoxon Stat	Wilcoxon *p*
Headband	2.49 (12)	0.028	[0.42, 6.20]	dz = 0.69, gz = 0.65	0.238	14.5	0.033
Fp1	1.31 (12)	0.215	[−1.68, 6.76]	dz = 0.36, gz = 0.34	0	14	0.167
Fp2	−0.66 (12)	0.521	[−6.94, 3.71]	dz = −0.18, gz = −0.17	0.404	23	0.373
F7	1.34 (12)	0.205	[−1.68, 7.07]	dz = 0.37, gz = 0.35	0.419	23	0.206
F8	0.85 (12)	0.414	[−2.30, 5.22]	dz = 0.23, gz = 0.22	0.002	24	0.720
T7	2.59 (12)	0.024	[1.31, 15.15]	dz = 0.72, gz = 0.67	0.001	0	0.003
T8	1.50 (12)	0.160	[−2.49, 13.42]	dz = 0.41, gz = 0.39	0.117	15	0.108
O1	3.23 (12)	0.007	[3.60, 18.55]	dz = 0.90, gz = 0.84	0.079	4	0.002
O2	4.41 (12)	0.001	[5.10, 15.06]	dz = 1.22, gz = 1.14	0.050	0	0

**Table 5:** Comparison of comfort ratings between male and female participants across overall headband and electrode sites.

Comparison	t (df)	*t*-Test *p*	95% CI	Effect	Shapiro *p*	Levene *p*	U Stat	Mann–Whitney *p*
Headband	1.69 (24)	0.105	[−0.88, 8.70]	d = 0.63, g = 0.61	0.608/0.009	0.455	111.5	0.101
Fp1	0.36 (24)	0.720	[−6.03, 8.60]	d = 0.14, g = 0.13	0.033/0.010	0.558	81.5	0.958
Fp2	0.07 (24)	0.946	[−6.14, 6.57]	d = 0.03, g = 0.03	0.113/0.146	0.896	79.5	1.000
F7	0.93 (24)	0.361	[−4.12, 10.89]	d = 0.35, g = 0.34	0.246/0.225	0.432	93.5	0.492
F8	1.00 (24)	0.327	[−3.37, 9.72]	d = 0.37, g = 0.36	0.354/0.023	0.442	91.0	0.579
T7	−0.79 (24)	0.439	[−13.51, 6.01]	d = −0.34, g = −0.33	0.228/0.256	0.519	67.0	0.509
T8	−0.96 (24)	0.349	[−13.24, 4.82]	d = −0.41, g = −0.39	0.410/0.198	0.580	63.5	0.399
O1	−0.01 (24)	0.989	[−10.98, 10.83]	d = −0.01, g = −0.01	0.481/0.564	0.475	79.0	0.979
O2	−0.43 (24)	0.669	[−12.19, 7.96]	d = −0.17, g = −0.16	0.545/0.479	0.583	73.5	0.751

**Table 6:** Sleep stage statistics in HBSleep.

Sleep Stage	Epoch Count	Percentage
Wake	4353	19.68%
N1	1456	6.58%
N2	9855	44.55%
N3	3116	14.09%
REM	3340	15.10%
Total	22,120	100%

**Table 7:** Average performance results (%) of EEG channels from the HBSleep dataset on three baseline models.

Model	Accuracy	Precision	Recall	F1 Score	Kappa Coefficient
DeepSleepNet	73.89 ± 1.42	63.78 ± 3.04	63.45 ± 2.95	62.37 ± 2.72	63.21 ± 1.63
TinySleepNet	74.81 ± 1.35	64.13 ± 3.20	63.48 ± 3.36	62.33 ± 2.82	63.28 ± 1.83
AttnSleepNet	75.81 ± 0.92	64.58 ± 3.14	64.73 ± 3.41	62.70 ± 2.94	65.52 ± 1.49

**Table 8:** Performance summary and comparison of wearable devices for sleep monitoring.

Systems	EEG Channels	EEG Electrode Type	Input Noise	EEG In Hair Region	Multi-Modal	Flexible	Plastic Housing	Sleep Monitoring
Kwon 2023—Patches [19]	2	Dry	Not specified	No	Yes	Yes	No	Yes
Kartsch 2019—BioWolf [16]	8	Wet	Not specified	Yes	No	No	Yes	No
Wang 2024—Headband [17]	6	Dry	Not specified	No	No	Yes	No	Yes
Kim 2020—Headband [18]	1	Wet	Not specified	No	Yes	Yes	Yes	Yes
Lin 2017—Forehead EEG [20]	5	Dry	Not specified	No	No	Yes	Yes	Yes
Han 2024—EarSleep [14]	0	None	Not specified	No	Yes	No	Yes	Yes
Cognionics Headset [15]	8	Dry	<1 µV RMS	Yes	No	Yes	Yes	No
This Work	8	Dry	0.24 µV RMS	Yes	Yes	Yes	No	Yes

## Data Availability

Data is unavailable due to privacy or ethical restrictions.
